# Correction to “COCOMO2: A Coarse-Grained Model for Interacting Folded and Disordered Proteins”

**DOI:** 10.1021/acs.jctc.5c02061

**Published:** 2025-12-30

**Authors:** Alexander Jussupow, Divya Bartley, Lisa J. Lapidus, Michael Feig

The acknowledgments are updated to reflect a missing grant (MCB2210228) that supported the research in the published paper:

