# Supplementary material for: COCOMO2: A Coarse-Grained Model for Interacting Folded and Disordered Proteins
Source: J Chem Theory Comput. 2025 Feb 5;21(4):2095–107. doi: 10.1021/acs.jctc.4c01460 (PMC11866933; doi:10.1021/acs.jctc.4c01460)
Supplement: Supplementary file 1 — ct4c01460_si_001.pdf [file ct4c01460_si_001.pdf]

SUPPLEMENTARY INFORMATION

COCOMO2: A coarse-grained model for interacting folded and disordered proteins

Alexander Jussupow[1], *Divya Bartley*[1], *Lisa J. Lapidus*[2], *Michael Feig*[1*]

[1]Department of Biochemistry and Molecular Biology, [2]Department of Physics and Astronomy
Michigan State University, East Lansing, MI 48824, USA

*Corresponding author
Michael Feig
603 Wilson Road, Room 218 BCH
East Lansing, MI 48824, USA
mfeiglab@gmail.com
+1-517-432-7439

Tables S1-S11

Figures S1-S10

Supplementary References

Table S1. Multi-domain proteins used for testing of COCOMO2 and the effect of λ.

Protein	Rg [nm]	N	Folded domains	Ions [M]	pH	Reference
THB_C2	1.91	137	[6, 42] [50, 137]	0.15	6.5	Michie *et al.* [1]
Ubq2	2.19	162	[11, 82] [87, 158]	0.33	8.0	Jussupow *et al.*[2]
Ubq3	2.62	228	[1, 72] [77, 148] [153, 224]	0.33	8.0	Jussupow *et al.*[2]
Gal3	2.91	250	[117, 250]	0.04	7.0	Lin *et al.*[3]
TIA1	2.75	275	[6, 82] [95, 172] [190, 275]	0.10	6.0	Sonntag *et al.*[4]
Ubq4	3.19	304	[1, 72] [77, 148] [153, 224] [229, 300]	0.33	8.0	Jussupow *et al.*[2]
hnRNPA1	3.12	314	[11, 89] [105, 179]	0.15	7.5	Martin *et al.*[5]
hSUMO_hnRNPA1	3.37	433	[44, 114] [132, 209] [224, 298]	0.10	7.5	Martin *et al.*[5]
GS0	3.2	470	[1, 226] [256, 470]	0.15	7.4	Moses *et al.*[6]
GS8	3.37	486	[1, 226] [272, 486]	0.15	7.4	Moses *et al.*[6]
GS16	3.45	502	[1, 226] [288, 502]	0.15	7.4	Moses *et al.*[6]
GS24	3.57	518	[1, 226] [304, 518]	0.15	7.4	Moses *et al.*[6]
GS32	3.75	534	[1, 226] [320, 534]	0.15	7.4	Moses *et al.*[6]
GS48	4.11	566	[1, 226] [352, 566]	0.15	7.4	Moses *et al.*[6]
SH4UD_SH3_SH2	3.28	264	[94, 150] [166, 258]	0.216	8.0	Gurumoorty *et al.*[7]
H46	4.15	381	[140, 355]	0.163	6.5	Elena-Real *et al.*[8]
TDP43W2A	4.11	415	[5, 77] [107, 177] [193, 260] [321, 329]	0.312	8.0	Wright *et al.*[9]
PCPE	4.04	424	[12, 125] [134, 249] [293, 412]	0.506	7.4	Bernocco *et al.*[10]
NiV_V	6.97	457	[406, 457]	0.232	8.0	Salladini *et al.*[11]
HeV_V	6.86	458	[404, 456]	0.232	8.0	Salladini *et al.*[11]
D14	3.9	483	[31, 121] [157, 246] [265, 354] [400, 479]	0.156	7.5	Hajizadeh *et al.*[12]
S4FL	4.7	552	[15, 138] [287 542]	0.169	7.2	Gomes *et al.*[13]
ChiAM	4.73	682	[8, 89] [92, 172] [178, 257] [266, 356] [359, 462] [471, 567] [578, 668]	0.282	8.0	Mazurkewich *et al.*[14]

Table S2. Amino acid-specific reference surface areas (S_{ref}) used in COCOMO2.

Residue	S_{ref} [nm^2]
ALA	0.796
ARG	1.921
ASN	1.281
ASP	1.162
CYS	1.074
GLN	1.575
GLU	1.462
GLY	0.544
HIS	1.634
ILE	1.410
LEU	1.519
LYS	1.923
MET	1.620
PHE	1.869
PRO	0.974
SER	0.933
THR	1.128
TRP	2.227
TYR	2.018
VAL	1.232

Table S3. RLP phase separation hysteresis simulation set.

Protein	N_{chain} (cond)	N_{chain} (free)	c [µM]	Box [nm]
RLP	0	180	1.0	669
RLP	0	180	2.0	531
RLP	0	180	5.0	391
RLP	0	180	7.5	342
RLP	0	180	10.0	310
RLP	0	180	12.5	288
RLP	0	180	15.0	271
RLP	0	180	20.1	246
RLP	0	180	24.9	229
RLP	0	180	30.1	215
RLP	0	180	35.2	204
RLP	0	180	39.7	196
RLP	0	180	59.8	171
RLP	0	180	90.4	149
RLP	0	180	118.8	136
RLP	180	0	1.0	669
RLP	180	0	2.0	531
RLP	180	0	5.0	391
RLP	180	0	7.5	342
RLP	180	0	10.0	310
RLP	180	0	12.5	288
RLP	180	0	15.0	271
RLP	180	0	20.1	246
RLP	180	0	24.9	229
RLP	180	0	30.1	215
RLP	180	0	35.2	204
RLP	180	0	39.7	196
RLP	180	0	59.8	171
RLP	180	0	90.4	149
RLP	180	0	118.8	136

Table S4. Homotypic protein phase separation systems for IDPs and multi-domain proteins.

Protein	$c_{sat,\,exp}$ [μM]	N_{chain} (cond)	N_{chain} (free)	Box [nm]	Folded domains	Reference
hTau40-k18	40	229	227	104		Ambadipudi *et al.*[15]
A1 LCD	125	217	77	101		Bremer *et al.*[16]
LAF1	24	178	81	177		Elbaum-Garfinkle *et al.*[17]
RLP	3	180	15	171		Dai *et al.*[18]
FUS LCD	235	172	35	69		Kaur *et al.*[19]
αSyn	500	0	300	100		Ray *et al.*[20]
hnRNPA1	173	200	77	90	[11, 89] [105, 179]	Martin *et al.*[5]
hSUMO_hnRNPA1	136	200	60	90	[44, 114] [132, 209] [224, 298]	Martin *et al.*[5]
MAGOH	110	300	6	50	[1 146]	Golovanov *et al.*[21]
Ref2NM	150	170	30	80	[87 166]	Golovanov *et al.*[21]
Y14	340	142	58	63	[71 149]	Golovanov *et al.*[21]
TAP	40	69	31	100	[118 194] [204 355] [380 548] [564 619]	Golovanov *et al.*[21]
GFP FUS	4.9	100	14	168	[286 368] [423 451] [529 755]	Wang *et al.*[22]
WW34	420	400	600	85	[15 40] [59 82]	Golovanov *et al.*[21]

Table S5. Parameter sets used to establish the link between potential energy and saturation concentration for IDPs and multi-domain proteins.

Protein	ε_{polar}	$\varepsilon_{charged}$	$\varepsilon_{hydrophobic}$	$A_{0,polar}$	$A_{0,hydrophobic}$	λ	Epot [kL/(mol · N)]	$Log_{10}(c_{sat})$
hTau40-k18	0.40		0.50	0.07	0		1.548	2.063
hTau40-k18	0.40		0.41	0.052	0		1.463	1.348
hTau40-k18	0.40		0.41	0.054	0		1.498	1.652
hTau40-k18	0.40		0.41	0.056	0		1.533	2.027
A1 LCD	0.40		0.41	0.05	0		1.448	0.225
A1 LCD	0.40	0.35	0.41	0.05	0		1.462	0.484
A1 LCD	0.40	0.30	0.41	0.05	0		1.478	0.694
A1 LCD	0.40	0.25	0.41	0.05	0		1.495	0.933
A1 LCD	0.40	0.20	0.41	0.05	0		1.514	1.051
A1 LCD	0.375		0.41	0.05	0		1.51	1.048
A1 LCD	0.35		0.41	0.05	0		1.573	1.786
A1 LCD	0.40		0.50	0.08	0		1.619	2.243
A1 LCD	0.40		0.50	0.07	0		1.466	0.776
A1 LCD	0.38	0.325	0.41	0.05	0		1.496	0.86
LAF1	0.40		0.50	0.06	0		1.452	0.125
LAF1	0.40		0.50	0.07	0		1.612	1.556
LAF1	0.385	0.325	0.41	0.05	0		1.594	1.262
LAF1	0.38	0.325	0.41	0.05	0		1.605	1.423
LAF1	0.40		0.41	0.05	0		1.518	0.392
LAF1	0.40	0.35	0.41	0.05	0		1.55	0.731
LAF1	0.40	0.30	0.41	0.05	0		1.585	1.132
LAF1	0.40	0.25	0.41	0.05	0		1.622	1.68
LAF1	0.375		0.41	0.05	0		1.587	1.261
LAF1	0.40	0.3875	0.41	0.05	0		1.56	0.678
LAF1	0.40	0.375	0.41	0.05	0		1.603	1.434
RLP	0.3875	0.3875	0.41	0.05	0		1.398	0.167
RLP	0.375	0.375	0.41	0.05	0		1.444	0.718
RLP	0.3625	0.3625	0.41	0.05	0		1.49	1.329
RLP	0.40		0.41	0.05	0		1.353	-0.734
RLP	0.40	0.35	0.41	0.05	0		1.39	-0.165
RLP	0.40	0.30	0.41	0.05	0		1.43	0.525
RLP	0.40	0.25	0.41	0.05	0		1.473	0.977
RLP	0.375		0.41	0.05	0		1.426	0.343
RLP	0.35		0.41	0.05	0		1.499	1.287
RLP	0.39	0.325	0.41	0.05	0		1.418	-0.065
RLP	0.38	0.325	0.41	0.05	0		1.429	0.334
RLP	0.37	0.325	0.41	0.05	0		1.439	0.675
RLP	0.40		0.50	0.05	0		1.443	0.65
RLP	0.40		0.50	0.06	0		1.398	0.167
RLP	0.40		0.50	0.07	0		1.444	0.718
FUS LCD	0.40		0.50	0.06	0		1.741	1.322
FUS LCD	0.39	0.325	0.41	0.05	0		1.789	1.971
FUS LCD	0.38	0.325	0.41	0.05	0		1.817	2.356
FUS LCD	0.40	0.3875	0.41	0.05	0		1.808	2.259
FUS LCD	0.40	0.36	0.41/0.47*	0.05	0		1.779	1.917
FUS LCD	0.40	0.32	0.41/0.53*	0.05	0		1.793	2.034
FUS LCD	0.40		0.41	0.052	0		1.806	2.255
FUS LCD	0.40		0.41	0.054	0		1.841	2.63
αSyn	0.20		0.443	0.025	0.0098		1.792	2.285
αSyn	0.238		0.45	0.022	0.0075		1.569	-0.067
αSyn	0.175		0.443	0.022	0.0098		1.83	2.606
αSyn	0.238		0.38	0.022	0.0098		1.794	2.348
αSyn	0.238		0.443	0.027	0.015		1.798	2.346
αSyn	0.22		0.43	0.022	0.0098		1.718	1.517
αSyn	0.238		0.443	0.024	0.098		1.659	0.964
hnRNPA1	0.35		0.41	0.05	0	0.50	3.695	1.821
hnRNPA1	0.35		0.41	0.05	0	0.40	3.661	1.511
hnRNPA1	0.39	0.325	0.41	0.05	0	0.40	3.624	0.486
hnRNPA1	0.39	0.325	0.41	0.05	0	0.50	3.659	1.125
hnRNPA1	0.40	0.30	0.41	0.053	0	0.40	3.676	1.633
hnRNPA1	0.40	0.30	0.41	0.053	0	0.30	3.592	0.549
hnRNPA1	0.40	0.30	0.41	0.053	0	0.20	3.706	2.196

hnRNPA1	0.238		0.442	0.022	0.098	0.60	3.682	1.672
hnRNPA1	0.238		0.443	0.022	0.098	0.60	3.682	1.631
hnRNPA1	0.216		0.479	0.031	0.0014	0.51	3.676	1.509
hSUMO_hnRNPA1	0.35		0.41	0.05	0	0.50	3.621	1.649
hSUMO_hnRNPA1	0.35		0.41	0.05	0	0.40	3.585	1.271
hSUMO_hnRNPA1	0.39	0.325	0.41	0.05	0	0.40	3.55	0.144
hSUMO_hnRNPA1	0.39	0.325	0.41	0.05	0	0.50	3.588	0.68
hSUMO_hnRNPA1	0.40	0.30	0.41	0.053	0	0.40	3.606	1.346
hSUMO_hnRNPA1	0.40	0.30	0.41	0.053	0	0.30	3.634	2.097
hSUMO_hnRNPA1	0.40	0.30	0.41	0.053	0	0.20	3.604	1.194
hSUMO_hnRNPA1	0.238		0.443	0.022	0.098	0.60	3.615	1.282
hSUMO_hnRNPA1	0.216		0.479	0.031	0.0014	0.51	3.605	1.24
hSUMO_hnRNPA1	0.238		0.442	0.022	0.098	0.51	5.74	1.649
MAGOH	0.39	0.325	0.41	0.05	0	0.30	5.709	2.08
MAGOH	0.39	0.325	0.41	0.05	0	0.25	5.675	1.676
MAGOH	0.39	0.325	0.41	0.05	0	0.20	5.597	1.388
MAGOH	0.40		0.41	0.05	0	0.20	5.667	0.78
MAGOH	0.40		0.41	0.05	0	0.30	5.73	1.107
MAGOH	0.40		0.41	0.05	0	0.40	5.79	1.813
MAGOH	0.40		0.41	0.05	0	0.50	5.74	2.407
Ref2NM	0.40		0.408	0.041	0.013	0.70	3.54	1.019
Ref2NM	0.216		0.479	0.031	0.0014	0.51	3.554	1.435
Ref2NM	0.267		0.453	0.028	0.095	0.60	3.535	1.319
Ref2NM	0.25		0.431	0.022	0.01	0.60	3.539	1.238
Ref2NM	0.238		0.443	0.022	0.098	0.60	3.55	1.349
Ref2NM	0.439		0.418	0.054	0.0086	0.58	3.527	0.571
Y14	0.40		0.408	0.041	0.013	0.70	3.588	0.404
Y14	0.216		0.479	0.031	0.0014	0.51	3.692	1.697
Y14	0.267		0.453	0.028	0.095	0.60	3.624	1.148
Y14	0.25		0.431	0.022	0.01	0.60	3.618	1.276
Y14	0.238		0.443	0.022	0.098	0.60	3.636	1.064
Y14	0.439		0.418	0.054	0.0086	0.58	3.6	0.318

Table S6. Heterotypic protein phase separation systems.

Protein	N_{chains}	Conc [μM]	Box [nm]	LLPS in sim	Length [μs]	LLPS Reference
FUS LCD/ $(RGRGG)_5$	266/266	201/201	130	No	10	Kaur *et al.*[19]
FUS LCD/ $(RGRGG)_5$	266/532	201/402	130	No	10	Kaur *et al.*[19]
FUS LCD/ $(RGRGG)_5$	266/1330	201/1005	130	Yes	10	Kaur *et al.*[19]
FUS LCD/ $(RGRGG)_5$	266/2660	201/2011	130	Yes	10	Kaur *et al.*[19]

Table S7. Protein – RNA phase separation systems.

Protein	N_{chains}	Conc [μM]	Box [nm]	LLPS in sim	Length [μs]	LLPS Reference
polyAde-21 / $(RRLR)_6$-SSSGSS	126/147	210/240	100	Yes	10	Bai *et al.*[23]
polyUra-40 / FUS LCD_{RGG3}	197/696	330/1160	100	Yes	4	Kaur *et al.*[19]
polyUra-10 / polyArg-50	360/72	600/120	100	Yes	10	Fisher & Elbaum-Garfinkle *et al.*[24]
polyAde-500 / $(RGRGG)_5$	30/1786	6/357	200	No	10	Alshareedah *et al.*[25]

Table S8. Analysis of system size effects.

System	N[a]	Box size [nm]	$c_{sat,sim}$ [μM]	σ[b]
Ref2NM	100	65	70	20
Ref2NM	150	73	48	14
Ref2NM	200	80	52	14
Ref2NM (preformed)	200	80	46	10
Ref2NM	300	92	64	15
FUS LCD	100	55	91	29
FUS LCD	150	62	64	22
FUS LCD	207	69	67	20
FUS LCD (preformed)	207	69	68	15
FUS LCD	300	82	55	15
hTau40	100	65	-	-
hTau40	150	75	161	27
hTau40	200	82	-	-
hTau40	300	80	191	27
hTau40 (preformed)	456	104	190	14
hTau40	456	104	207	19

[a]number of chains in the simulation box size
[b]standard deviation across the last 2 μs of a 5 μs simulation with COCOMO2.

Table S9. Linear fit parameters for relating interaction energy to log(c_{sat}) according to Eq. 9

Protein	a	b
hTau40-k18	8.88	-11.64
A1 LCD	11.51	-16.34
LAF1	12.44	-18.53
RLP	14.86	-20.82
FUS LCD	13.19	-21.61
αSyn	10.32	-16.21
hnRNPA1	20.96	-75.51
hSUMO_hnRNPA1	29.82	-106.30
MAGOH	8.37	-46.10
Ref2NM	33.00	-115.86
Y14	12.38	-43.99

Table S10. Convergence of saturation estimates.

System	$c_{sat,sim}$ [μM][a]	σ_{mean}[b]	σ[c]
hTau40-k18	190	2.6	14
A1 LCD	71	5.4	12
LAF1	27.2	1.9	3.5
RLP	4.5	0.3	0.6
FUS LCD	69.5	1.3	16
αSyn	23.4	2.3	5.7
hnRNPA1	30.1	1.2	7.6
hSUMO_hnRNPA1	4.4	0.6	4.3
MAGOH	1170	23	138
Ref2NM	46.1	2.0	11
Y14	5.10	0.8	6.2
TAP	0.1	0.1	0.4
GFP FUS	0.7	0.04	0.2
WW34	1191	17	67

[a]average solute concentration
[b]standard deviation of the mean (determined by block averaging)
[c]standard deviation over the last 2μs of the production run.

Table S11. IDP R_g test set

Protein	N	Rg [nm]	Reference
angiotensin	8	0.79	Ohnishi *et al.*[26]
ak16	16	0.98	Kohn *et al.*[27]
Hist5	24	1.38	Cragnell *et al.*[28]
CspTm	67	1.47	Müller-Späth *et al.*[29]
CTD2	83	2.61	Gibbs *et al.*[30]
erm	122	3.96	Lens *et al.*[31]
A1	137	2.76	Bremer *et al.*[16]
sNase	141	2.12	Flanagan *et al.*[32]
fhua	142	3.34	Riback *et al.*[33]
hTau-k25	185	4.1	Mylonas *et al.*[34]
CAHSD	229	4.8	Hesgrove *et al.*[35]
hTau-k27	231	3.7	Mylonas *et al.*[34]
PNt	334	5.1	Bowman *et al.*[36]
hTau-k25	450	4.1	Mylonas *et al.*[34]

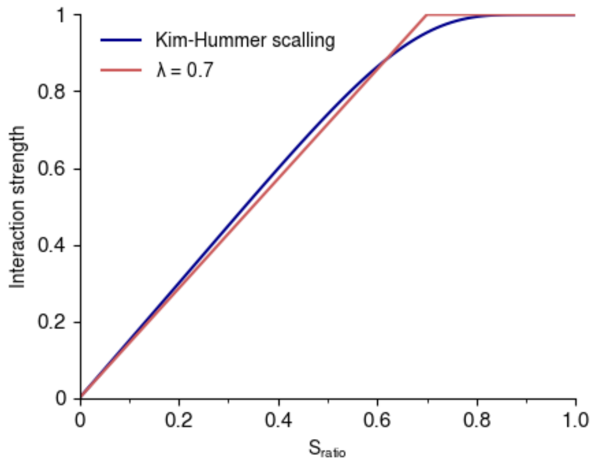

Figure S1. Solvent accessibility scaling function. The figure compares the surface scaling with λ = 0.7, with the sigmoid function form used by Kim *et al.*[37] (blue). Both approaches modulate interaction strength as a function of the solvent accessibility ratio (S_{ratio}), and can produce similar dependencies despite differences in functional form.

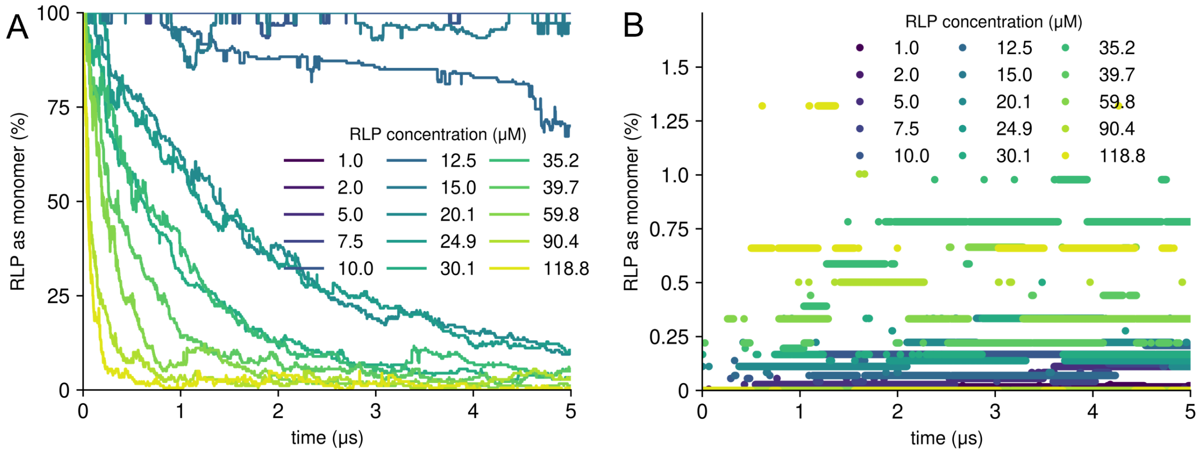

Figure S2. Time evolution of RLP monomer fraction across various concentrations. (A) and (B) show the percentage of RLP remaining as monomers over time at concentrations ranging from 1.0 μM to 118.8 μM starting from a randomly distributed (A) or condensate (B) state. Higher concentrations lead to faster depletion of monomers, indicating accelerated condensation.

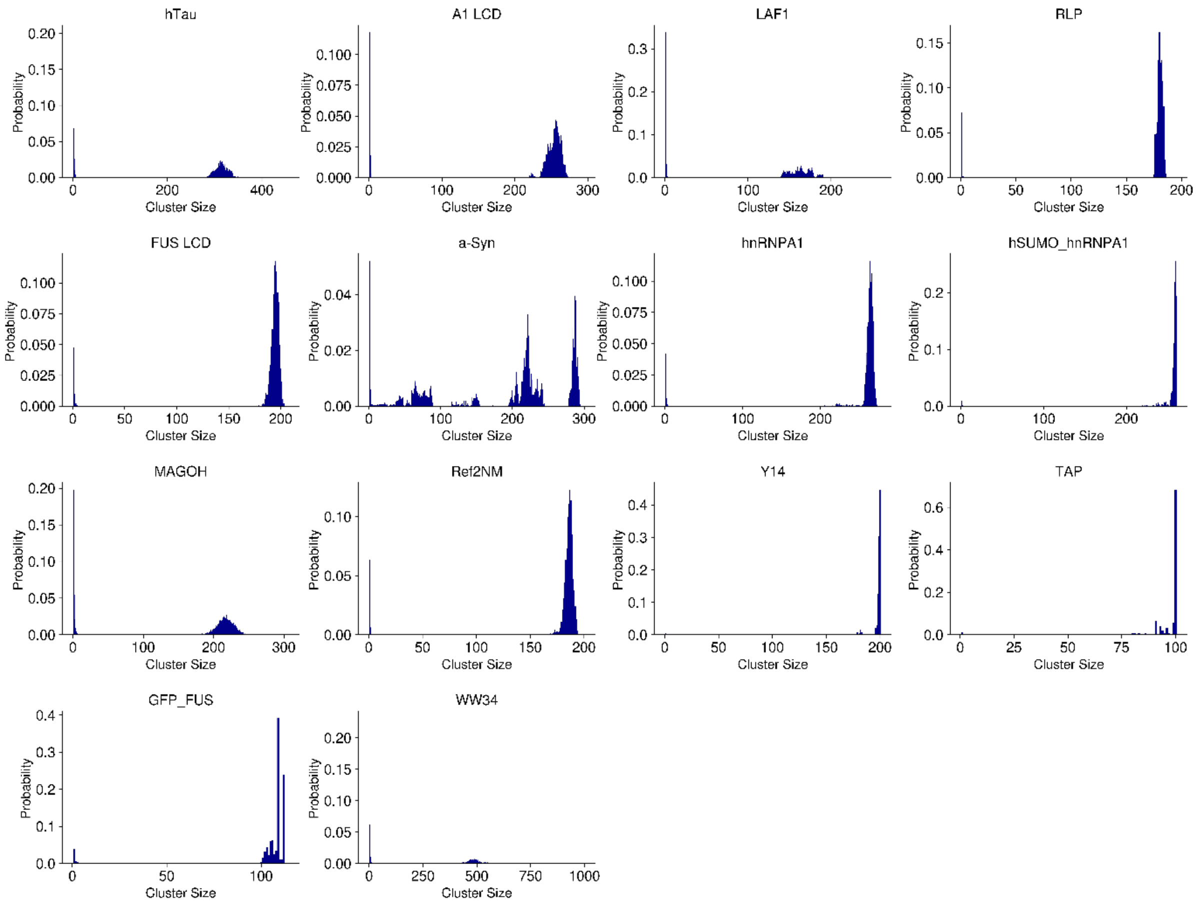

Figure S3. Cluster size distribution across all tested systems. The figure illustrates the cluster size distributions for tested systems (listed in Table S4) during the simulation. The cluster size was determined by a hierarchical clustering algorithm based on center-center distances. The clear separation between small and large clusters demonstrates phase coexistence, with the larger clusters representing the condensed phase and smaller clusters corresponding to the solute phase.

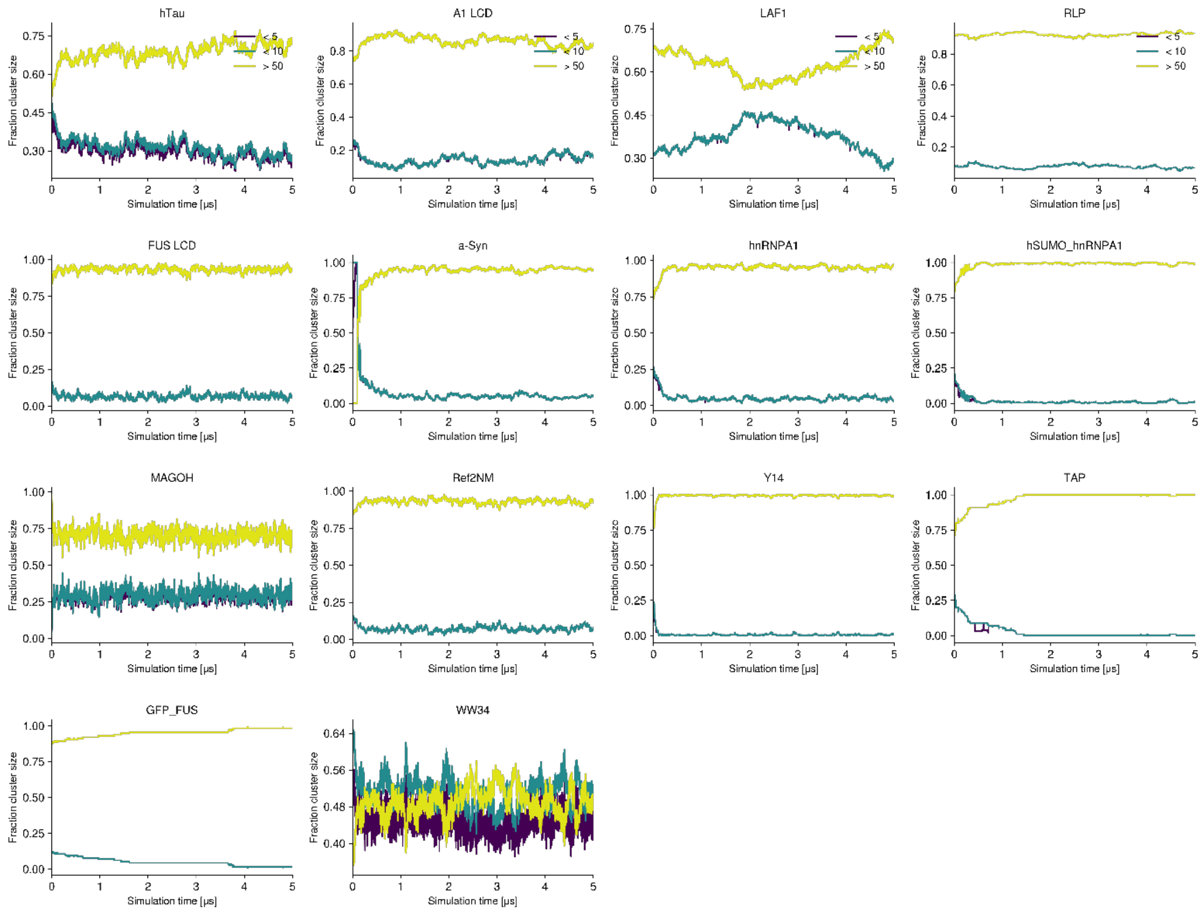

Figure S4. Time evolution of cluster sizes in simulated systems. The figure shows the fraction of clusters belonging to three size categories (<5, <10, and >50 molecules) over simulation time for the tested systems. For most systems, clusters with more than 50 molecules dominate the condensed phase, while smaller clusters (<5) represent the dilute phase. High-concentration systems, such as WW34, exhibit increased transient clustering, necessitating a higher threshold to distinguish the solute phase.

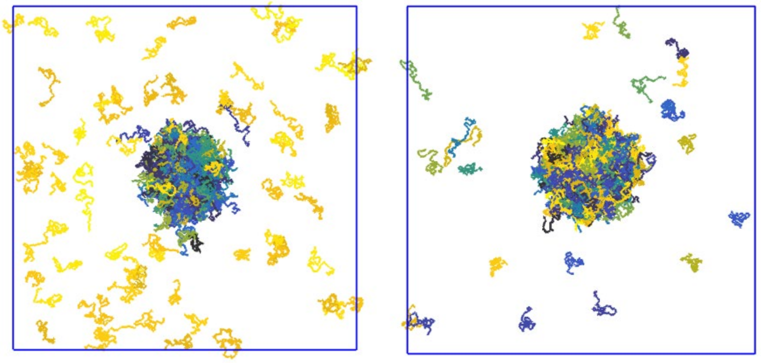

Figure S5. Initial and final frames of A1 LCD simulation. The figure shows the starting configuration (left) and the final configuration (right) for the A1 LCD simulation aimed at determining the saturation concentration (c_{sat}). The chains are colored based on the chain number. The initial frame features a preformed condensate surrounded by additional molecules in the dilute phase.

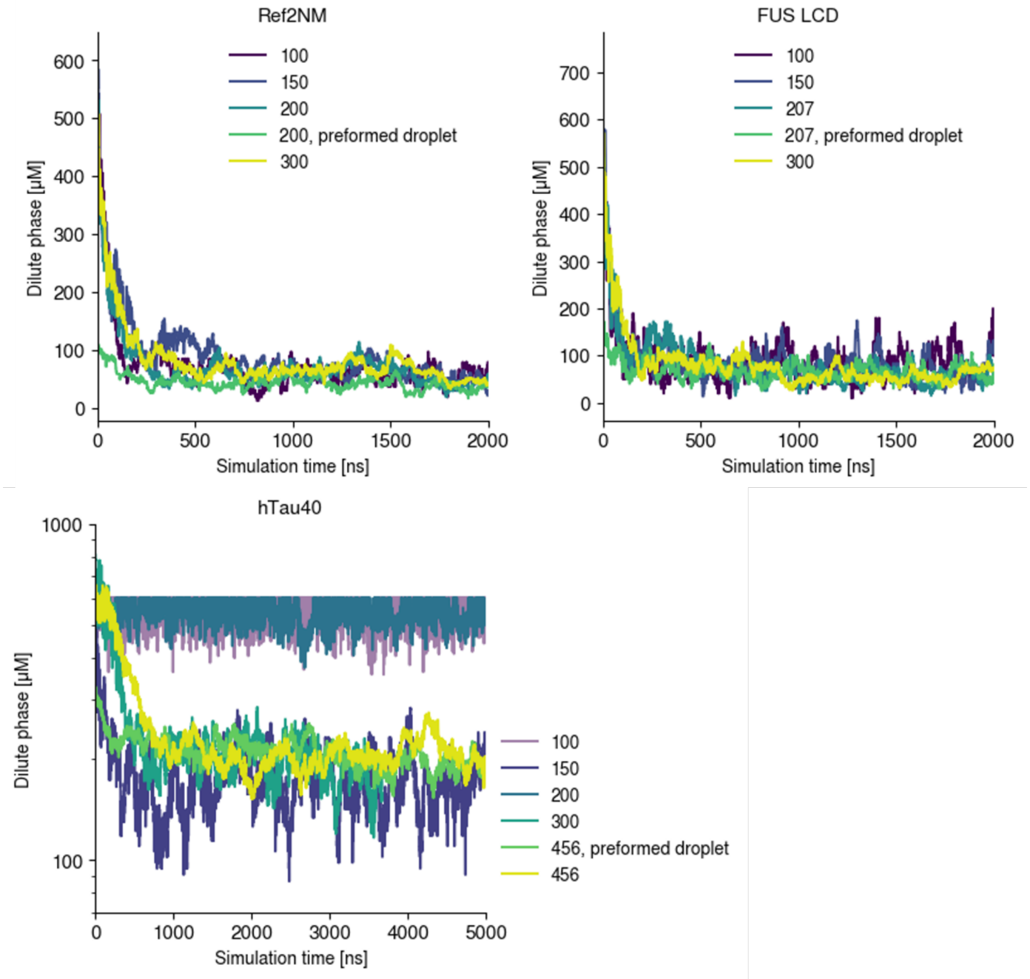

Figure S6. Dilute phase concentrations over time for varying initial configurations. The figure illustrates the time evolution of dilute phase concentrations for Ref2NM (top left), FUS LCD (top right), and hTau40 (bottom) across simulations with varying numbers of chains and initial setups. Simulations were conducted with fully diluted starting configurations (e.g., 100, 150, 200 chains) and preformed condensates (e.g., 200 or 456 chains, "preformed droplet"). For Ref2NM and FUS LCD, the dilute phase concentrations converge to similar values, regardless of the starting configuration. In contrast, hTau40 demonstrates unreliable condensation behavior at lower chain counts, with no condensation observed for setups with 100 or 200 chains near the csat.

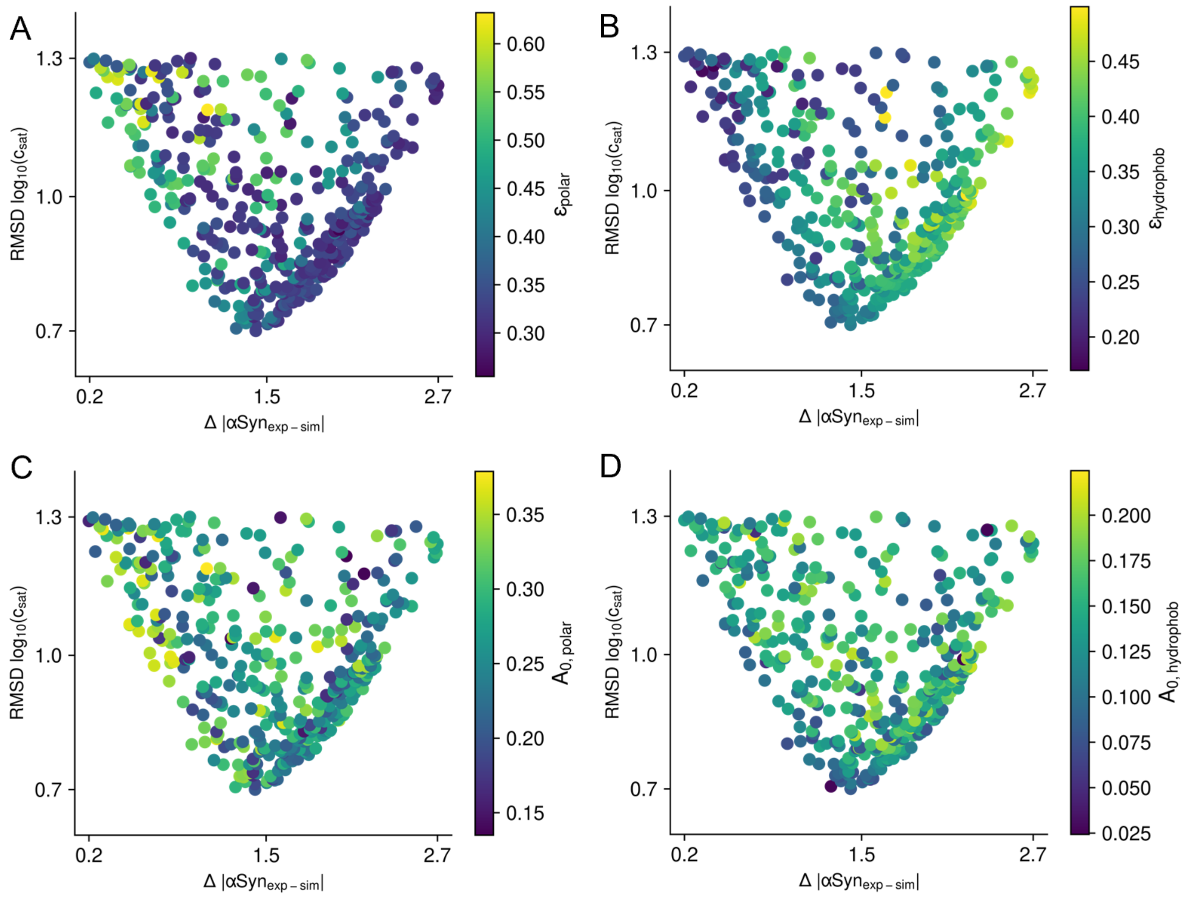

Figure S7. Exploration of parameter space for COCOMO2. (A-D) Comparison of RMSD deviation log10(c_{sat}) for IDPs and the log10(c_{sat}) deviation of αSyn from experimental values across 400 top-performing parameter sets. The points are colored based on the values of ε_{polar} (A), $\varepsilon_{hydrophobic}$ (B), $A_{0,polar}$ (C) and $A_{0,hydrophobic}$ (D).

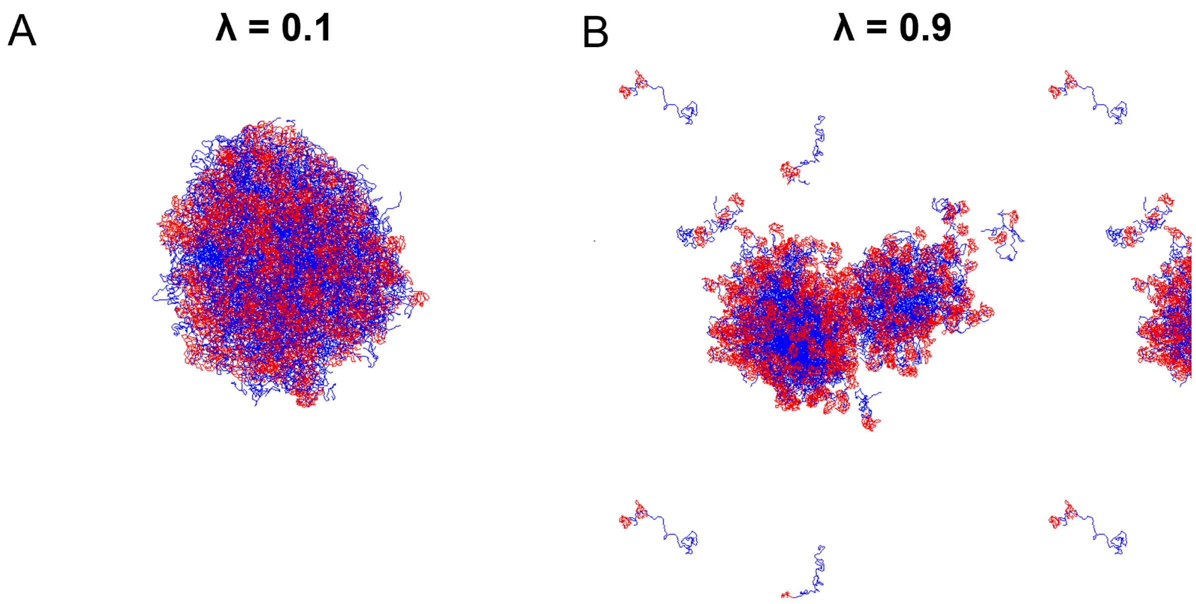

Figure S8. Morphological change of hnRNPA1 condensates for different values of λ. (A) At λ = 0.1, the folded domains are incorporated in the condensate, resulting in a dense structure. (B) At λ = 0.9, the folded domains become more flexible and are found at the surface of the condensate.

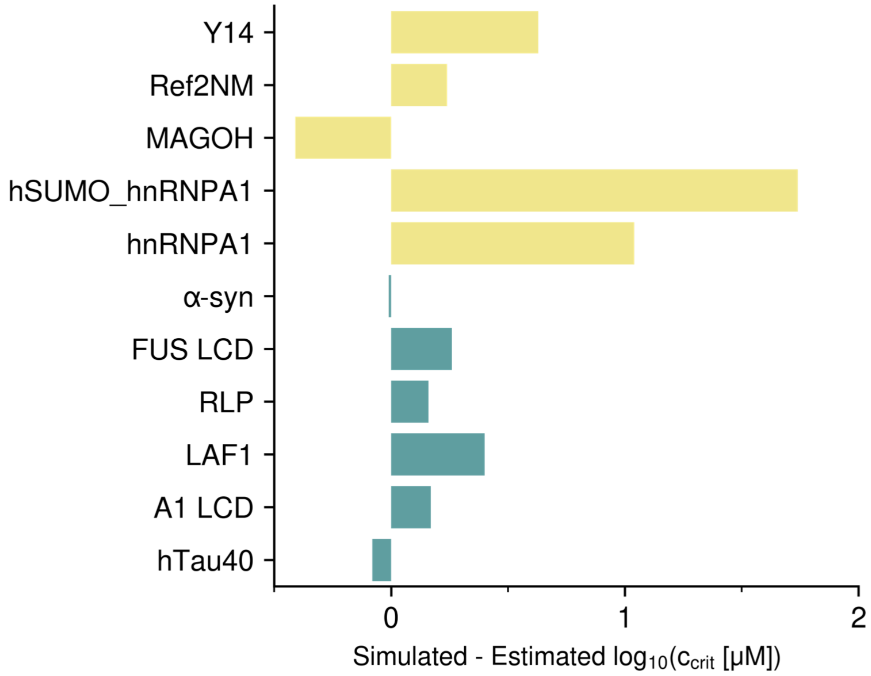

Figure S9. Comparison of the difference between simulated and estimated $\log_{10}(c_{crit})$ values for IDPs and multi-domain proteins. Proteins with smaller deviations are mostly IDPs (shown in blue), while larger deviations, particularly exceeding 1 log unit, are primarily observed for multi-domain proteins (in yellow).

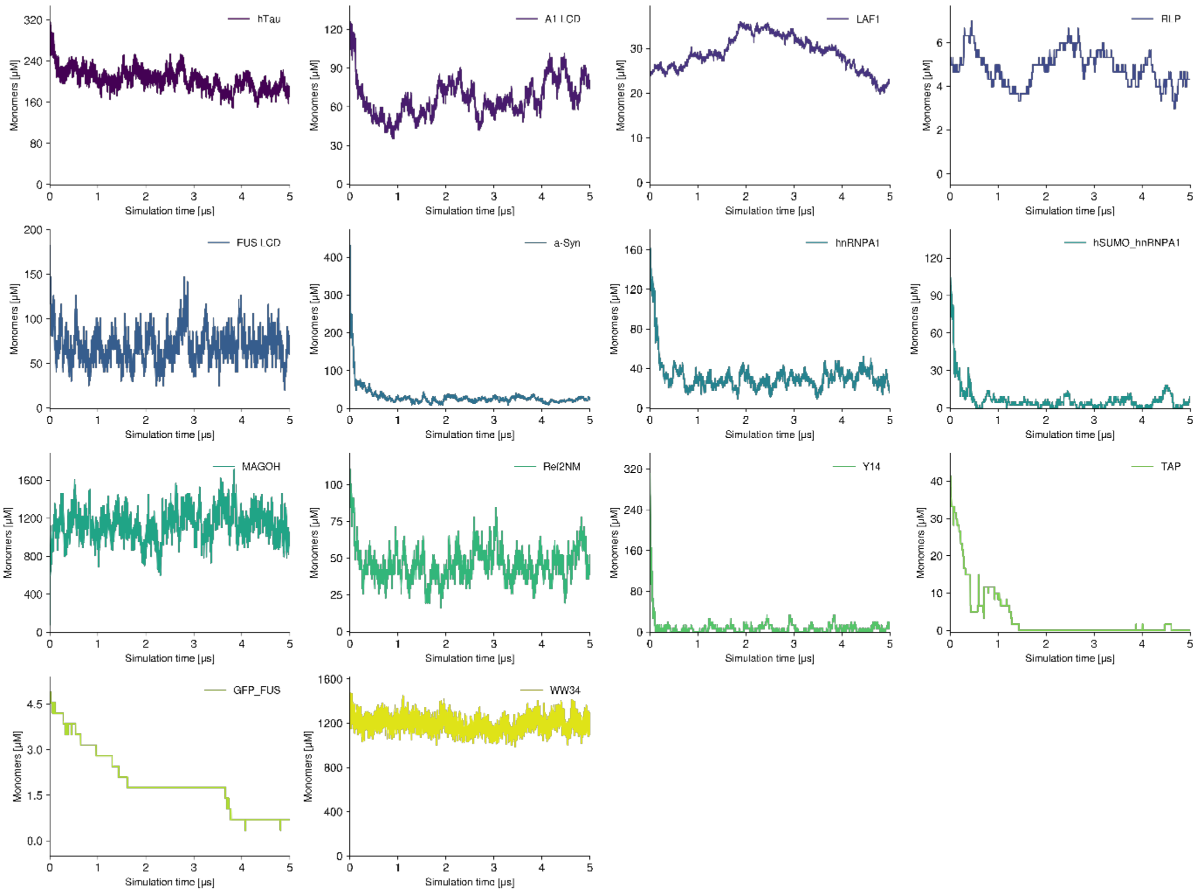

Figure S10. Time evolution of monomer concentration across training and test systems for IDPs and multi-domain proteins.

Supplementary References

(1) Michie, K. A.; Kwan, A. H.; Tung, C.-S.; Guss, J. M.; Trewhella, J. A Highly Conserved Yet Flexible Linker Is Part of a Polymorphic Protein-Binding Domain in Myosin-Binding Protein C. *Structure* **2016**, *24* (11), 2000–2007. https://doi.org/10.1016/j.str.2016.08.018.

(2) Jussupow, A.; Messias, A. C.; Stehle, R.; Geerlof, A.; Solbak, S. M. Ø.; Paissoni, C.; Bach, A.; Sattler, M.; Camilloni, C. The Dynamics of Linear Polyubiquitin. *Sci Adv* **2020**, *6* (42). https://doi.org/10.1126/sciadv.abc3786.

(3) Lin, Y.-H.; Qiu, D.-C.; Chang, W.-H.; Yeh, Y.-Q.; Jeng, U.-S.; Liu, F.-T.; Huang, J. The Intrinsically Disordered N-Terminal Domain of Galectin-3 Dynamically Mediates Multisite Self-Association of the Protein through Fuzzy Interactions. *Journal of Biological Chemistry* **2017**, *292* (43), 17845–17856. https://doi.org/10.1074/jbc.M117.802793.

(4) Sonntag, M.; Jagtap, P. K. A.; Simon, B.; Appavou, M.; Geerlof, A.; Stehle, R.; Gabel, F.; Hennig, J.; Sattler, M. Segmental, Domain-Selective Perdeuteration and Small-Angle Neutron Scattering for Structural Analysis of Multi-Domain Proteins. *Angewandte Chemie International Edition* **2017**, *56* (32), 9322–9325. https://doi.org/10.1002/anie.201702904.

(5) Martin, E. W.; Thomasen, F. E.; Milkovic, N. M.; Cuneo, M. J.; Grace, C. R.; Nourse, A.; Lindorff-Larsen, K.; Mittag, T. Interplay of Folded Domains and the Disordered Low-Complexity Domain in Mediating HnRNPA1 Phase Separation. *Nucleic Acids Res* **2021**, *49* (5), 2931–2945. https://doi.org/10.1093/nar/gkab063.

(6) Moses, D.; Guadalupe, K.; Yu, F.; Flores, E.; Perez, A. R.; McAnelly, R.; Shamoon, N. M.; Kaur, G.; Cuevas-Zepeda, E.; Merg, A. D.; Martin, E. W.; Holehouse, A. S.; Sukenik, S. Structural Biases in Disordered Proteins Are Prevalent in the Cell. *Nat Struct Mol Biol* **2024**. https://doi.org/10.1038/s41594-023-01148-8.

(7) Gurumoorthy, V.; Shrestha, U. R.; Zhang, Q.; Pingali, S. V.; Boder, E. T.; Urban, V. S.; Smith, J. C.; Petridis, L.; O'Neill, H. Disordered Domain Shifts the Conformational Ensemble of the Folded Regulatory Domain of the Multidomain Oncoprotein C-Src. *Biomacromolecules* **2023**, *24* (2), 714–723. https://doi.org/10.1021/acs.biomac.2c01158.

(8) Elena-Real, C. A.; Sagar, A.; Urbanek, A.; Popovic, M.; Morató, A.; Estaña, A.; Fournet, A.; Doucet, C.; Lund, X. L.; Shi, Z.-D.; Costa, L.; Thureau, A.; Allemand, F.; Swenson, R. E.; Milhiet, P.-E.; Crehuet, R.; Barducci, A.; Cortés, J.; Sinnaeve, D.; Sibille, N.; Bernadó, P. The Structure of Pathogenic Huntingtin Exon 1 Defines the Bases of Its Aggregation Propensity. *Nat Struct Mol Biol* **2023**, *30* (3), 309–320. https://doi.org/10.1038/s41594-023-00920-0.

(9) Wright, G. S. A.; Watanabe, T. F.; Amporndanai, K.; Plotkin, S. S.; Cashman, N. R.; Antonyuk, S. V.; Hasnain, S. S. Purification and Structural Characterization of Aggregation-Prone Human TDP-43 Involved in Neurodegenerative Diseases. *iScience* **2020**, *23* (6), 101159. https://doi.org/10.1016/j.isci.2020.101159.

(10) Bernocco, S.; Steiglitz, B. M.; Svergun, D. I.; Petoukhov, M. V.; Ruggiero, F.; Ricard-Blum, S.; Ebel, C.; Geourjon, C.; Deléage, G.; Font, B.; Eichenberger, D.; Greenspan, D. S.; Hulmes, D. J. S. Low Resolution Structure Determination Shows Procollagen C-

Proteinase Enhancer to Be an Elongated Multidomain Glycoprotein. *Journal of Biological Chemistry* **2003**, *278* (9), 7199–7205. https://doi.org/10.1074/jbc.M210857200.

(11) Salladini, E.; Delauzun, V.; Longhi, S. The Henipavirus V Protein Is a Prevalently Unfolded Protein with a Zinc-Finger Domain Involved in Binding to DDB1. *Mol. BioSyst.* **2017**, *13* (11), 2254–2267. https://doi.org/10.1039/C7MB00488E.

(12) Hajizadeh, N. R.; Pieprzyk, J.; Skopintsev, P.; Flayhan, A.; Svergun, D. I.; Löw, C. Probing the Architecture of a Multi-PDZ Domain Protein: Structure of PDZK1 in Solution. *Structure* **2018**, *26* (11), 1522-1533.e5. https://doi.org/10.1016/j.str.2018.07.016.

(13) Gomes, T.; Martin-Malpartida, P.; Ruiz, L.; Aragón, E.; Cordeiro, T. N.; Macias, M. J. Conformational Landscape of Multidomain SMAD Proteins. *Comput Struct Biotechnol J* **2021**, *19*, 5210–5224. https://doi.org/10.1016/j.csbj.2021.09.009.

(14) Mazurkewich, S.; Helland, R.; Mackenzie, A.; Eijsink, V. G. H.; Pope, P. B.; Brändén, G.; Larsbrink, J. Structural Insights of the Enzymes from the Chitin Utilization Locus of Flavobacterium Johnsoniae. *Sci Rep* **2020**, *10* (1), 13775. https://doi.org/10.1038/s41598-020-70749-w.

(15) Ambadipudi, S.; Biernat, J.; Riedel, D.; Mandelkow, E.; Zweckstetter, M. Liquid–Liquid Phase Separation of the Microtubule-Binding Repeats of the Alzheimer-Related Protein Tau. *Nat Commun* **2017**, *8* (1), 275. https://doi.org/10.1038/s41467-017-00480-0.

(16) Bremer, A.; Farag, M.; Borcherds, W. M.; Peran, I.; Martin, E. W.; Pappu, R. V.; Mittag, T. Deciphering How Naturally Occurring Sequence Features Impact the Phase Behaviours of Disordered Prion-like Domains. *Nat Chem* **2022**, *14* (2), 196–207. https://doi.org/10.1038/s41557-021-00840-w.

(17) Elbaum-Garfinkle, S.; Kim, Y.; Szczepaniak, K.; Chen, C. C.-H.; Eckmann, C. R.; Myong, S.; Brangwynne, C. P. The Disordered P Granule Protein LAF-1 Drives Phase Separation into Droplets with Tunable Viscosity and Dynamics. *Proc. Natl. Acad. Sci. USA* **2015**, *112* (23), 7189–7194. https://doi.org/10.1073/pnas.1504822112.

(18) Dai, Y.; Farag, M.; Lee, D.; Zeng, X.; Kim, K.; Son, H.; Guo, X.; Su, J.; Peterson, N.; Mohammed, J.; Ney, M.; Shapiro, D. M.; Pappu, R. V.; Chilkoti, A.; You, L. Programmable Synthetic Biomolecular Condensates for Cellular Control. *Nat Chem Biol* **2023**, *19* (4), 518–528. https://doi.org/10.1038/s41589-022-01252-8.

(19) Kaur, T.; Raju, M.; Alshareedah, I.; Davis, R. B.; Potoyan, D. A.; Banerjee, P. R. Sequence-Encoded and Composition-Dependent Protein-RNA Interactions Control Multiphasic Condensate Morphologies. *Nat Commun* **2021**, *12* (1), 872. https://doi.org/10.1038/s41467-021-21089-4.

(20) Ray, S.; Singh, N.; Kumar, R.; Patel, K.; Pandey, S.; Datta, D.; Mahato, J.; Panigrahi, R.; Navalkar, A.; Mehra, S.; Gadhe, L.; Chatterjee, D.; Sawner, A. S.; Maiti, S.; Bhatia, S.; Gerez, J. A.; Chowdhury, A.; Kumar, A.; Padinhateeri, R.; Riek, R.; Krishnamoorthy, G.; Maji, S. K. α-Synuclein Aggregation Nucleates through Liquid–Liquid Phase Separation. *Nat Chem* **2020**, *12* (8), 705–716. https://doi.org/10.1038/s41557-020-0465-9.

(21) Golovanov, A. P.; Hautbergue, G. M.; Wilson, S. A.; Lian, L.-Y. A Simple Method for Improving Protein Solubility and Long-Term Stability. *J Am Chem Soc* **2004**, *126* (29), 8933–8939. https://doi.org/10.1021/ja049297h.

(22) Wang, J.; Choi, J.-M.; Holehouse, A. S.; Lee, H. O.; Zhang, X.; Jahnel, M.; Maharana, S.; Lemaitre, R.; Pozniakovsky, A.; Drechsel, D.; Poser, I.; Pappu, R. V.; Alberti, S.; Hyman, A. A. A Molecular Grammar Governing the Driving Forces for Phase Separation of Prion-like RNA Binding Proteins. *Cell* **2018**, *174* (3), 688-699.e16. https://doi.org/10.1016/j.cell.2018.06.006.

(23) Bai, Q.; Zhang, Q.; Jing, H.; Chen, J.; Liang, D. Liquid–Liquid Phase Separation of Peptide/Oligonucleotide Complexes in Crowded Macromolecular Media. *J Phys Chem B* **2021**, *125* (1), 49–57. https://doi.org/10.1021/acs.jpcb.0c09225.

(24) Fisher, R. S.; Elbaum-Garfinkle, S. Tunable Multiphase Dynamics of Arginine and Lysine Liquid Condensates. *Nat Commun* **2020**, *11* (1), 4628. https://doi.org/10.1038/s41467-020-18224-y.

(25) Alshareedah, I.; Kaur, T.; Ngo, J.; Seppala, H.; Kounatse, L.-A. D.; Wang, W.; Moosa, M. M.; Banerjee, P. R. Interplay between Short-Range Attraction and Long-Range Repulsion Controls Reentrant Liquid Condensation of Ribonucleoprotein–RNA Complexes. *J Am Chem Soc* **2019**, *141* (37), 14593–14602. https://doi.org/10.1021/jacs.9b03689.

(26) Ohnishi, S.; Kamikubo, H.; Onitsuka, M.; Kataoka, M.; Shortle, D. Conformational Preference of Polyglycine in Solution to Elongated Structure. *J Am Chem Soc* **2006**, *128* (50), 16338–16344. https://doi.org/10.1021/ja066008b.

(27) Kohn, J. E.; Millett, I. S.; Jacob, J.; Zagrovic, B.; Dillon, T. M.; Cingel, N.; Dothager, R. S.; Seifert, S.; Thiyagarajan, P.; Sosnick, T. R.; Hasan, M. Z.; Pande, V. S.; Ruczinski, I.; Doniach, S.; Plaxco, K. W. Random-Coil Behavior and the Dimensions of Chemically Unfolded Proteins. *Proc. Natl. Acad. Sci. USA* **2004**, *101* (34), 12491–12496. https://doi.org/10.1073/pnas.0403643101.

(28) Cragnell, C.; Durand, D.; Cabane, B.; Skepö, M. Coarse-grained Modeling of the Intrinsically Disordered Protein Histatin 5 in Solution: Monte Carlo Simulations in Combination with SAXS. *Proteins: Structure, Function, and Bioinformatics* **2016**, *84* (6), 777–791. https://doi.org/10.1002/prot.25025.

(29) Müller-Späth, S.; Soranno, A.; Hirschfeld, V.; Hofmann, H.; Rüegger, S.; Reymond, L.; Nettels, D.; Schuler, B. Charge Interactions Can Dominate the Dimensions of Intrinsically Disordered Proteins. *Proc. Natl. Acad. Sci. USA* **2010**, *107* (33), 14609–14614. https://doi.org/10.1073/pnas.1001743107.

(30) Gibbs, E. B.; Lu, F.; Portz, B.; Fisher, M. J.; Medellin, B. P.; Laremore, T. N.; Zhang, Y. J.; Gilmour, D. S.; Showalter, S. A. Phosphorylation Induces Sequence-Specific Conformational Switches in the RNA Polymerase II C-Terminal Domain. *Nat Commun* **2017**, *8* (1), 15233. https://doi.org/10.1038/ncomms15233.

(31) Lens, Z.; Dewitte, F.; Monté, D.; Baert, J.-L.; Bompard, C.; Sénéchal, M.; Van Lint, C.; de Launoit, Y.; Villeret, V.; Verger, A. Solution Structure of the N-Terminal Transactivation Domain of ERM Modified by SUMO-1. *Biochem Biophys Res Commun* **2010**, *399* (1), 104–110. https://doi.org/10.1016/j.bbrc.2010.07.049.

(32) Flanagan, J. M.; Kataoka, M.; Shortle, D.; Engelman, D. M. Truncated Staphylococcal Nuclease Is Compact but Disordered. *Proc. Natl. Acad. Sci. USA* **1992**, *89* (2), 748–752. https://doi.org/10.1073/pnas.89.2.748.

(33) Riback, J. A.; Bowman, M. A.; Zmyslowski, A. M.; Knoverek, C. R.; Jumper, J. M.; Hinshaw, J. R.; Kaye, E. B.; Freed, K. F.; Clark, P. L.; Sosnick, T. R. Innovative Scattering Analysis Shows That Hydrophobic Disordered Proteins Are Expanded in Water. *Science (1979)* **2017**, *358* (6360), 238–241. https://doi.org/10.1126/science.aan5774.

(34) Mylonas, E.; Hascher, A.; Bernadó, P.; Blackledge, M.; Mandelkow, E.; Svergun, D. I. Domain Conformation of Tau Protein Studied by Solution Small-Angle X-Ray Scattering. *Biochemistry* **2008**, *47* (39), 10345–10353. https://doi.org/10.1021/bi800900d.

(35) Hesgrove, C. S.; Nguyen, K. H.; Biswas, S.; Childs, C. A.; Shraddha, K. C.; Medina, B. X.; Alvarado, V.; Yu, F.; Sukenik, S.; Malferrari, M.; Francia, F.; Venturoli, G.; Martin, E. W.; Holehouse, A. S.; Boothby, T. C. Tardigrade CAHS Proteins Act as Molecular Swiss Army Knives to Mediate Desiccation Tolerance Through Multiple Mechanisms. *bioRxiv* **2021**. https://doi.org/10.1101/2021.08.16.456555.

(36) Bowman, M. A.; Riback, J. A.; Rodriguez, A.; Guo, H.; Li, J.; Sosnick, T. R.; Clark, P. L. Properties of Protein Unfolded States Suggest Broad Selection for Expanded Conformational Ensembles. *Proc. Natl. Acad. Sci. USA* **2020**, *117* (38), 23356–23364. https://doi.org/10.1073/pnas.2003773117.

(37) Kim, Y. C.; Hummer, G. Coarse-Grained Models for Simulations of Multiprotein Complexes: Application to Ubiquitin Binding. *J Mol Biol* **2008**, *375* (5), 1416–1433. https://doi.org/10.1016/j.jmb.2007.11.063.
